# Ranolazine-Functionalized Copper Nanoparticles as a Colorimetric Sensor for Trace Level Detection of As^3+^

**DOI:** 10.3390/nano9010083

**Published:** 2019-01-10

**Authors:** Gul Naz Laghari, Ayman Nafady, Sameerah I. Al-Saeedi, Sirajuddin, Syed Tufail H. Sherazi, Jan Nisar, Muhammad Raza Shah, Mohammad I. Abro, Munazza Arain, Suresh K. Bhargava

**Affiliations:** 1 National Centre of Excellence in Analytical Chemistry, University of Sindh, Jamshoro 76080, Pakistan; gulnaz.laghari@yahoo.com (G.N.L.); tufail.sherazi@gmail.com (S.T.H.S.); 2 Department of Chemistry, College of Science, King Saud University, Riyadh 11451, Saudi Arabia; 3 Department of Chemistry, Faculty of Science, Sohag University, Sohag 82524, Egypt; 4 Department of Chemistry, College of Science, Princess Nourah bint Abdulrahman University, Riyadh 11451, Saudi Arabia; sialsaeedi@pnu.edu.sa; 5 National Centre of Excellence in Physical Chemistry, University of Peshawar, Peshawar 25120, Pakistan; pashkalawati@gmail.com; 6 International Centre of Chemical and Biological Science, HEJ Research Institute of Chemistry, University of Karachi, Karachi 75270, Pakistan; raza.shah@iccs.edu; 7 Department of Metallurgy and Materials Engineering, Mehran University of Engineering & Technology, Jamshoro 76080, Pakistan; m_ishaqueabro@yahoo.com; 8 Dr. MA Kazi Institute of Chemistry, University of Sindh, Jamshoro 76080, Pakistan; munazzaarain2493@gmail.com; 9 Centre for Advanced Materials and Industrial Chemistry (CAMIC), School of Applied Sciences, RMIT University, GPO BOX 2476, Melbourne 3001, Australia; suresh.bhargava@rmit.edu.au

**Keywords:** ranolazine, copper nanoparticles, colorimetric sensor, arsenic, ground water

## Abstract

This study involves environmentally friendly synthesis of copper nanoparticles in aqueous medium without inert gas protection, using ranolazine as a capping material. UV-Visible (UV-Vis) spectrometry showed that ranolazine-derived copper nanoparticles (Rano-Cu NPs) demonstrate a localized surface plasmon resonance (LSPR) band at 573 nm with brick-red color under optimized parameters, including pH, reaction time, and concentrations of copper salt, hydrazine hydrate, and ranolazine. The coating of ranolazine on the surface of Cu NPs was studied via Fourier transform infrared (FTIR) spectroscopy. Scanning electron microscopy (SEM) revealed that Rano-Cu NPs consist of spherical particles. X-ray diffraction (XRD) verified that Rano-Cu NPs are crystalline in nature. Atomic force microscopy (AFM) showed that the average size of Rano-Cu NPs was 40 ± 2 nm in the range of 22–95 nm. Rano-Cu NPs proved to be highly sensitive as a selective colorimetric sensor for As^3+^ via color change from brick red to dark green, in the linear range of 3.0 × 10^−7^ to 8.3 × 10^−6^ M, with an R² value of 0.9979. The developed sensor is simple, cost effective, highly sensitive, and extremely selective for As^3+^ detection, showing a low detection limit (LDL) of 1.6 × 10^−8^ M. The developed sensor was effectively tested for detection of As^3+^ in some water samples.

## 1. Introduction

The contamination of drinking water and groundwater by arsenic (As^3+^) is one of the leading health issues in the world [1]. It is reported that around 140 million people around the world may have been exposed to drinking water contaminated with levels of As^3+^ higher than the World Health Organization (WHO) guideline of 10 ppb [2]. As^3+^ is broadly distributed in the Earth’s crust. Natural as well as anthropogenic origins, such as industrial discharge, weathering of rocks, ore mining, and meteorological deposits, have enhanced arsenic levels in the residue, soil, and water resources [3,4]. Exposure to arsenic can produce a variety of adverse health effects, including dermal changes and cardiovascular, gastrointestinal, respiratory, geotaxis, mutagenic, and carcinogenic effects [5]. Acute arsenic poisoning could result in dryness of mouth, nausea, and gastro-intestinal issues [6]. Even more dangerous is that arsenic cannot be eliminated from the body, accumulating in human tissue [7]. Arsenic occurs predominantly in the form of inorganic arsenic As^3+^, which is 60 times more poisonous than As^5+^ or organic arsenic compounds [8].

Currently, the conventional methods employed for arsenic detection include atomic absorption spectrometry (AAS) [9,10], inductively coupled plasma (ICP) absorption spectrometry [11,12], and high-performance liquid chromatography (HPLC) [13]. These methods have proven to be complicated, expensive, with non-portable instruments, and time-consuming protocols, so they are neither easily available in developing countries nor capable for on-site field detection. The electrochemical approach for determining As^3+^ with a low detection limit is well-known, but laboratory settings are still necessary. These can be obtained from several electrode studies based on several optimization studies and also from experience with the interference from the co-deposition of other metals [14]. Therefore, it is necessary to develop highly sensitive, time-saving, and capable of on-site and cost-effective methods.

As an alternative solution to the above-mentioned problems, metal nanoparticles (NPs) have witnessed tremendous growth due to their impressive optical, physical, and electrical qualities in modern ages, resulting in the mitigation of several complicated problems. Compared with costly silver, gold, and platinum NPs, the cost of forming copper nanoparticles (Cu NPs) is certainly lower [15]. However, the novel optoelectronic features associated with copper and silver as well as gold nanoparticles facilitate the use of these in various applications, such as sensors, catalysis, electronics, biomedicine, and bioanalytics [16].

In view of their tremendous applications, several facile procedures have been established to fabricate these metal nanoparticles at a controlled size, good shape, and better surface properties [17]. The formation of copper nanoparticles (Cu NPs) with high stability is quite challenging due to their possible oxidation in the water medium [18,19]. Considered more economical than gold and silver nanoparticles and possessing similar features, Cu NPs may be an effective replacement for gold and silver nanoparticles for the fabrication of a highly economical sensing probe. Although their economic benefits are encouraging and tremendously useful applications for Cu NPs have been found, the colorimetric sensing of Cu NPs is infrequently highlighted in comparison to gold and silver nanoparticles [20].

Numerous methods have been published based on chemical and physical strategies for the synthesis of Cu NPs. The microemulsion method is the most employed well-known chemical strategy, but it is considered costly due to the waste of a large amount of surfactant [21]. Other techniques used for making NPs, including aerosol, radiolysis, and laser ablation, involve the use of expensive instruments that incur waste of high power, reducing their demand [22]. Another strategy is to use the bubbling of inert gas for the fabrication of Cu NPs, but due to the formation of large particles, this method was also not employed [23]. Many researchers; thus, prefer to use hydrazine as a reductant as well as a nitrogen provider to remove oxygen from the water medium and, hence, avoid oxidation of the resulting Cu NPs.

Herein, we describe the formation of ranolazine-derived copper nanoparticles (Rano-Cu NPs) using hydrazine reduction or protection strategy where ranolazine acts as a capping compound. Moreover, to the best of our knowledge, there is no information available in the literature concerning the employment of Cu NPs as a colorimetric sensor for ultrasensitive detection of As^3+^. Thus, we believe that the current method is a cost effective and facile approach for As^3+^ detection at the trace level.

## 2. Experimental

### 2.1. Materials and Methods

Chemicals employed in this work were of the ultrapure analytical quality and were used as collected from supplier. All solutions were prepared in Milli-Q water. Copper(II) chloride pentahydrate (CuCl_2_·5H_2_O) and hydrazine monohydrate (N_2_H_4_·H_2_O) were purchased from Merck (Karachi, Pakistan), while ranolazine (C_24_H_33_N_3_O_4_) was obtained from Fluka Chemicals (Shanghai, China). Meanwhile, sodium hydroxide (NaOH) (98%), hydrochloric acid (HCl) (37%), and different salts, including diarsenic trioxide (As_2_O_3_), cadmium nitrate (Cd(NO_3_)_2_), lead nitrate (Pb(NO_3_)_2_), calcium chloride (CaCl_2_), cobalt nitrate (Co(NO_3_)_2_), iron(II) sulfate, hepta hydrate (FeSO_4_·7H_2_O), nickel(II) (NiSO_4_), sodium nitrate (NaNO_3_), zinc nitrate (Zn(NO_3_)_2_), and magnesium chloride (MgCl_2_), were purchased from Sigma-Aldrich (Karachi, Pakistan).

### 2.2. Instrumentation

Localized surface plasmon resonance (LSPR bands) of Rano-Cu NPs and other application studies were conducted via Perkin-Elmer Lambda 356 UV-Vis spectrophotometer from 200–800 nm. FTIR spectral studies of Cu NPs and ranolazine were conducted with the help of Nicolet 5700 spectrometer from Thermo Scientific (Beijing, China) using the KBr disc method, to verify the interaction of Cu NPs with ranolazine. Information regarding the shapes and sizes of Rano-Cu NPs was determined via atomic force microscopy (model 550, Agilent, Santa Clara, CA, USA) and scanning electron microscopy (model JSM-6380LV, JEOL Ltd., Tokyo, Japan). Crystalline features of Cu NPs were investigated by X-ray diffractometer (D-8 Bruker, Billerica, MA, USA). A high-quality Android mobile digital camera with better resolution power served to take colored photos of some reactions.

### 2.3. Preparation of Rano-Cu NPs

Rano-Cu NPs were prepared in a 10-mL capacity test tube. Accordingly, 400 µL of 1 M hydrazine, 500 µL of 1% ranolazine solution, and 300 µL of 0.02 M CuCl_2_ solution were mixed together. To this mixture, 5.8 mL of Milli-Q water was added, and the mixture was left for color development. After 25 min, the color of the solution changed from light brown to brick red. The pH of the entire solution was observed to be 8.3, and the color of Cu NPs was stable for several weeks. Hydrazine hydrate was used as the reducing agent, which was essential for the formation of Cu NPs, protecting them from oxidation in the aqueous media due to its nitrogen-releasing property.

### 2.4. Procedure for Colorimetric Detection of As^3+^ by Rano-Cu NPs

According to the procedure, each 3 mL of Rano-Cu NPs was treated by adding 10 µL of As^3+^ solution with different concentrations of As^3+^ to get a final range of 0.3 to 8.3 µM As^3+^. After 3–4 min, each solution was transferred into a quartz cell to record the UV-Vis response against a blank solution. The change in color from brick red to dark green was observed, allowing for visual discrimination of As^3+^ ion.

### 2.5. Detection of As^3+^ Ion in Environmental Water Samples

To validate the applicability of this work, the concentration of As^3+^ in ground water samples collected around Jamshoro, Hala, and Tandojam districts of Sindh Province, Pakistan was determined. These samples were filtered through a 0.22 µm mesh membrane and transferred to a vial before the final stage of the analytical assay. As the addition of a blank run resulted in no change to the Cu NP signal, the As^3+^ solution was made in the respective water sample. Then, an appropriate quantity of 10 µL of As^3+^ was mixed with 3 mL of Cu NPs, which caused a marked decrease in the signal intensity. The change in absorbance (∆A) values versus the added different concentrations of As^3+^, obtained in the calibration curve, was plotted to get a linear range.

## 3. Results and Discussion

### 3.1. Optical Characterization

The surface plasmon resonance (SPR) of Cu, Au, or Ag NPs exists within the optical part of the electromagnetic wavelength [24]. For the sizes between 10 to 60 nm, these NPs exhibit LSPR bands around 570 nm, 520 nm, and 400 nm, with red, purple, and yellow solutions, respectively [25,26,27]. It has also been observed that the size and geometrical shape of metal NPs have a great effect on their optical characteristics [28]. Optical spectroscopy could serve as a basic characterization tool to provide information about the formation of Rano-Cu NPs. Crucial parameters for Cu NP_S_ formation, such as the concentration of CuCl_2_, ranolazine, hydrazine hydrate, as well as the pH, were optimized by UV-Vis spectrometry. Significantly, extremely small-sized Rano-Cu NPs with longer time stability (having hypochromic-shifted spectra) were obtained after adding optimum concentrations of the precursor salt solution.

The visible spectra of the related results are illustrated in Figure S1a (Supplementary Material) based on the optimization of precursor copper salt from 100–450 µL of 0.02 M solution. However, at lower concentrations of Cu^2+^ ions, there is no presence of the LSPR band, which may be due to insufficient amount of added salt essential for formation of the nanoparticles. Higher concentrations of precursor salt; however, showed a bathochromic shift in the spectrum due to the presence of both small as well as bigger particles. Such behavior may be associated with the nucleation-growth by Cu^2+^ at inappropriate concentrations, while the optimum concentration could be responsible for smaller NP formation [29,30]. An amount of 300 µL of 0.02 M copper chloride was thus selected as the optimum concentration, as it produced a blue-shifted peak at 577 nm.

The UV-Vis spectra in Figure S1b show the optimization of the reducing agent (hydrazine) in the range of 100–600 µL from its 1 M solution, and suggest that the most blue-shifted wavelength at 575 nm, resulted after the addition of 400 µL, was chosen as the optimum concentration. The increasing concentration of the reducing agent above this value resulted in the noted bathochromic shift. Moreover, the UV-Vis spectra (Figure S1c) clearly indicate that increasing the concentration of ranolazine from 100 to 600 µL induces a marked change in the LSPR band, and causes a blue-shifted peak at 573 nm when 500 µL of the 1% capping agent is employed as the optimum volume.

Finally, the UV-Vis spectra showing the effect of pH on absorbance and λ_max_ are contained in Figure S1d for a pH ranging from 5 to 11. The SPR is evidently visible for pH from 5 to 11. The study reveals that the most blue-shifted surface plasmon absorption band of Cu NPs occurs at 573 nm at pH 8, compared to the red-shifted wavelengths by Cu NPs formed at other pH values.

**Figure 1:**
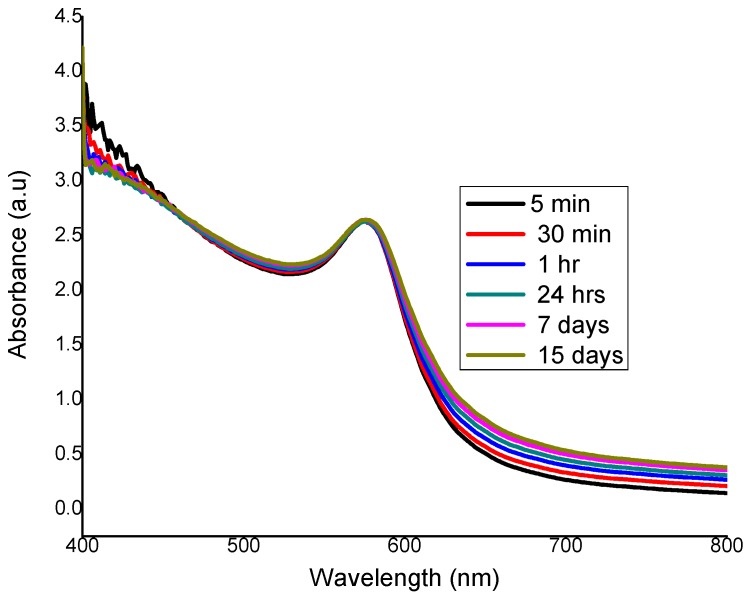
Time-dependent study of Rano-Cu NPs solution showing stability profile for a period of two weeks.

To check the stability of Rano-Cu NPs, the UV-Vis spectra were recorded at different time intervals (up to 15 days) after formation, as depicted in Figure 1. The data reveal that there was no variation in λ_max_ or color after more than two weeks. Previous work has demonstrated that Cu NPs remain stable in an inert environment. However, in the oxygen-rich environment present in the aqueous media, the LSPR intensity and concentration of Cu NPs gradually decrease because of oxidation [31].

### 3.2. FTIR Studies

To examine the interaction of Cu metal with ranolazine molecule, FTIR spectroscopy was conducted and Figure S2 contained the IR spectra recorded for pure ranolazine and Rano-Cu NPs, respectively. Various functional groups are present in ranolazine, showing characteristic signals depending on their distinct vibrational modes. It is observed in the spectrum (black curve) that bands at 3327 cm^−1^ and 1589 cm^−1^ can be assigned to (–NH) stretching and bending modes, respectively, whereas the 1681 cm^−1^ band confirms the presence of the carbonyl group (–C=O) of pure ranolazine. Additionally, peaks at 2933 and 1465 cm^−1^ are attributable to aliphatic and aromatic ring v(C–C) stretching and bending, respectively. On the other hand, IR spectrum obtained for Rano-Cu NPs (red curve) exhibited, overall, similar vibrational peaks; the peak due to the (–NH) group was much smaller, whereas the (–C=O) band disappeared and a new peak characteristic of Cu NPs appeared at 3440 cm^−1^. These few differences in peaks between the two spectra are indicative of the interaction between ranolazine molecules and Cu NPs, thereby eliminating the possibility of the formation of copper oxide [32,33,34].

### 3.3. Scanning Electron Microscopy (SEM) Analysis

**Figure 2:**
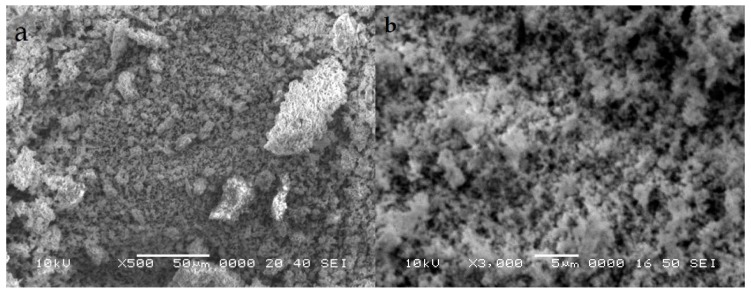
(**a**,**b**) SEM images of Rano-Cu NPs showing porous morphology with low and high magnification, respectively.

Morphological characteristics of the obtained Rano-Cu NPs were investigated by means of SEM microscopy, as shown in Figure 2a with low-resolution image and Figure 2b with high-resolution image, respectively. Figure 2b clearly illustrates that Rano-Cu NPs are small particles with wire-like interlinkages and porous texture, making them appropriate for special interaction with As^3+^ and, hence, selective sensing during application study.

### 3.4. Powder X-ray Diffraction (PXRD) Studies

The crystalline nature of Rano-Cu NPs was verified by PXRD diffraction. The results show that all the crystalline patterns of Rano-Cu NPs purely match with those of PDF data base card 040836. There was no peak to show impurities of CuO or Cu_2_O. The PXRD patterns (Figure S3) describe characteristic (111), (200), and (220) planes with face-centered cubic (fcc) structure observed at 2θ angle of 47.8, 53.3, and 69.7, respectively. Estimation of the crystallite size of Cu NPs resulted in an average size of 40 nm from the PXRD patterns, obtained by using the Scherrer’s equations displayed in Equation (1):

(1) $$D=\frac{K\lambda }{\beta Cos\theta }$$

where *D* represents the average size (nm), *K* is the Scherrer’s constant (0.9) describing the shape, *λ* is the wavelength of the X-ray (Cu Kα, 1.542 Å), *β* is the full-width at half-maximum intensity, and *θ* depicts the half of the diffraction peak angle [35]. The observation from the PXRD study is well-correlated with the values collected from AFM images.

### 3.5. Colorimetric Assay for As^3+^ Ions

**Figure 3:**
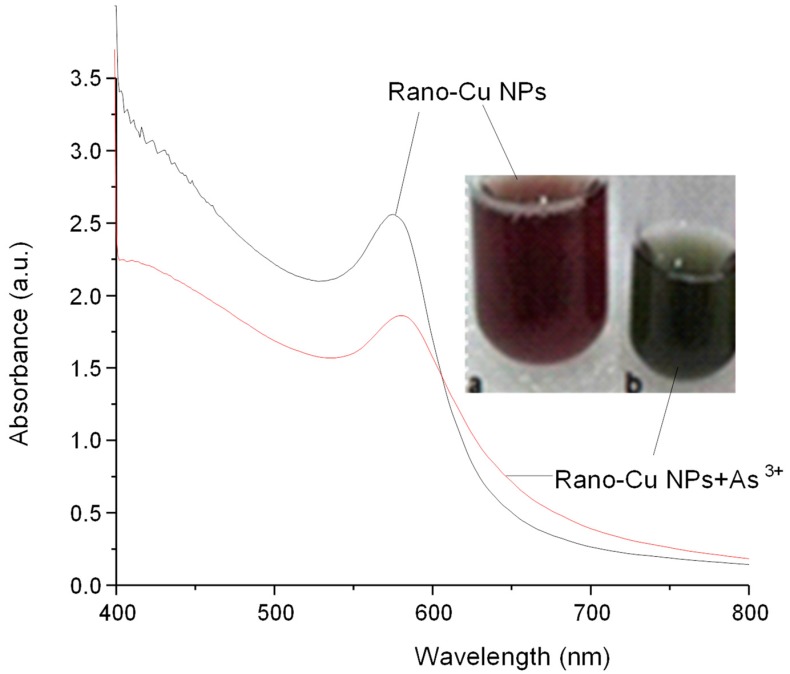
UV-Vis spectra for Rano-Cu NPs (black curve) and the interaction of Rano-Cu NPs with As^3+^ (red curve with 5 min reaction time). Inset shows color change according to UV-Vis spectral results.

To investigate the interaction of Rano-Cu NPs with As^3+^, a colorimetric variation from brick red to dark green was noticed upon addition of As^3+^ to Rano-Cu NPs (Figure 3). The respective spectrum of Rano-Cu NPs (black curve in Figure 3) showed a reduced absorption with a red-shifted wavelength after the addition of As^3+^, as can be seen from the red curve in Figure 3. Virtually, the addition of As^3+^ causes the removal of coated ranolazine from the NP surface, thereby causing the resulting Cu NPs to aggregate, as evidenced by the decrease in absorbance together with the red shift of the spectrum.

**Scheme 1:**
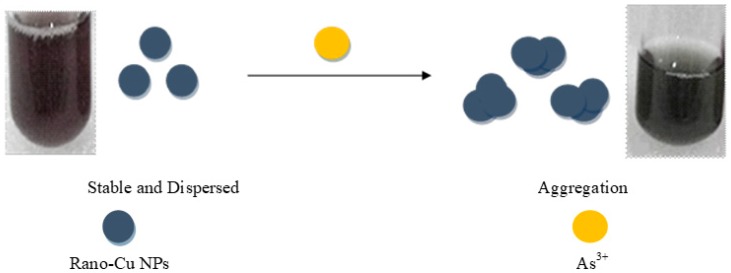
Proposed colorimetric aggregation-based sensing mechanism of As^3+^ via Rano-Cu NPs.

In light of the results from Figure 3, we can propose a mechanism (Scheme 1), which represents the interaction of As^3+^ with Cu NPs via the aggregation-based protocol.

### 3.6. AFM Studies

**Figure 4:**
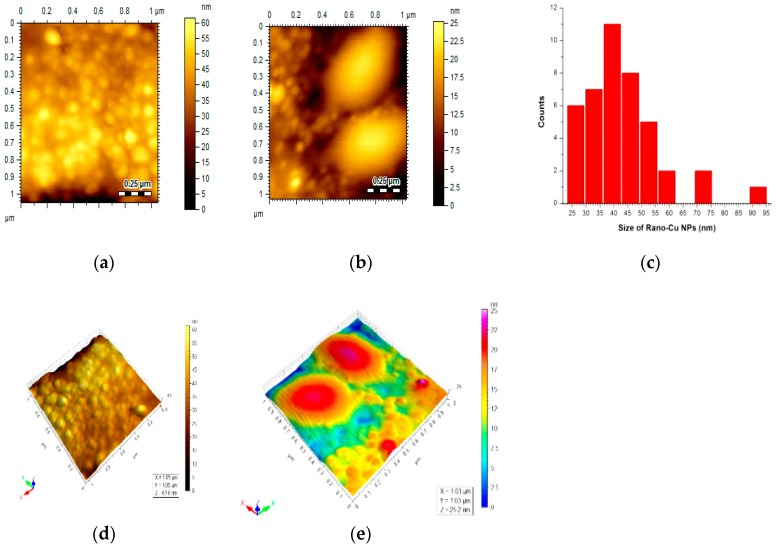
AFM image of (**a**) Rano-Cu NPs, (**b**) Rano-Cu NPs having 7-µM As^3+^, and (**c**) average size distribution bar diagram of Cu NPs (**a**) within the range of 22–95 nm. Parts (**d**) and (**e**) show the 3D topographical map of the (**a**) dispersed and (**b**) aggregated surface of Cu NPs.

AFM is one of the most powerful techniques for directly inspecting a 3D material surface at the nanometer scale. It does not require sophisticated sample preparation or facile to interpret, and provides information regarding the morphology of the material in a nondestructive manner [36]. The visual morphological change based on stable, dispersed, and aggregated nanoparticles can be easily investigated by the AFM imaging technique. It is evident from Figure 4a that the originally formed Rano-Cu NPs are spherical and monodisperse, possessing an average particle size of 40 ± 2 nm in the range of 22 to 95 nm, as shown in Figure 4c. The interaction of ranolazine-capped Cu NPs with As^3+^ (7 µM) depicts the formation of larger NPs aggregates (Figure 4b), while Figure 4d,e show the 3D topographical map of the dispersed and aggregated Cu NPs, respectively. According to AFM results, we consider that the change in size and LSPR signal is due to the removal of ranolazine from the surface of Rano-Cu NPs, which are then transformed into a big agglomeration of de-capped nanoparticles that cannot show surface plasmon, as described by Mie theory [37]. These AFM studies are in excellent agreement with the UV-Vis observations in Figure 3, as well as the proposed aggregation mechanism in Scheme 1.

### 3.7. Analytical Performance of the Cu NPs for Detecting As^3+^

**Figure 5:**
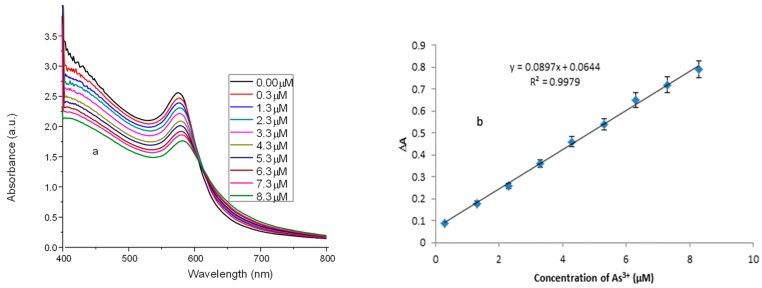
(**a**) Decrease in absorbance with increasing concentration of As^3+^. (**b**) Linear regression plot between ∆ absorbance and As^3+^ concentration.

Quantitative data were collected on the basis of addition of As^3+^ to Rano-Cu NPs in the UV-Vis spectral range of 200–800 nm (Figure 5a). The change in absorbance (∆A) versus concentration was then plotted to get the final result for a well-defined calibration plot. The sensor exhibited a highly linear response in the range from 3.0 × 10^−7^ to 8.3 × 10^−6^ M (Figure 5b). The correlation coefficient (R^2^) was 0.9979, with the limit of detection (LOD) and limit of quantification (LOQ) equal to 1.6 × 10^−8^ and 5.48 × 10^−8^ M, respectively. The LOD value was determined using 3 × σ/slope, where σ represents the standard deviation of three blank signals. The Rano-Cu NPs as a colorimetric sensor were further checked for long term usage, and it was noted that the sensor was active for As^3+^ detection with a relative standard deviation of 4.5%.

Table 1 presents the comparative data for As^3+^ detection by various reported sensors based on range, complication, and the limit of detection obtained by the present method and other techniques. Clearly, the Cu NPs used to detect As^3+^ in this work has a lower LOD, with simpler protocol, faster analysis, and a greater economical nature than other methods. Practically, the lower LOD reflects the better sensitivity of the developed sensor. Importantly, Table 1 further reveals that the use, by our group, of Cu NPs for colorimetric sensing of As^3+^ is the first study of its kind, which relies on the induced aggregation of Cu NPs in the presence of As^3+^ ions.

### 3.8. Selectivity of Developed Sensor

**Figure 6:**
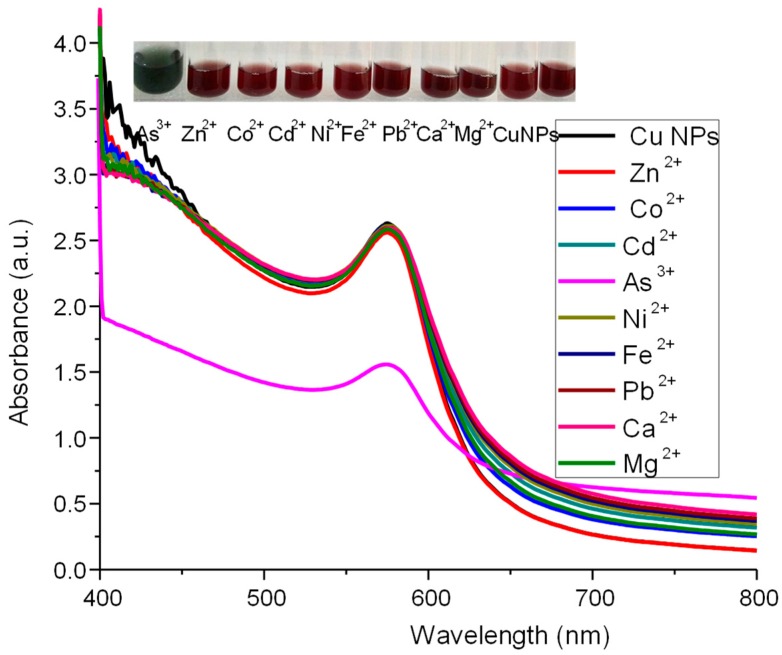
UV-Vis spectra showing the effect of adding As^3+^ and other metal ions to Rano-Cu NP solution; inset picture monitors the color change upon addition of mentioned ions to Rano-Cu NPs.

To check the selectivity of Rano-Cu NPs for As^3+^, we examined the response of Cu NPs for other metal ions such as Cd^2+^, Ni^2+^, Fe^2+^, Zn^2+^, Mg^2+^, Co^2+^, Ca^2+^, and Pb^2+^ at concentrations 10 times higher than that of As^3+^. Only the As^3+^-exposed Cu NP solution showed a color change from brick red to dark green, along with a decrease in absorbance and a small shift in wavelength, whereas there was negligible change in color and absorbance after the addition of other metal ions (Figure 6). This behavior confirms that our newly developed sensor is extremely selective regarding As^3+^ detection.

### 3.9. Application of Rano-Cu NPs for Detecting As^3+^ in Groundwater Samples

In this framework, three samples of the groundwater collected from different locations near Jamshoro, Hala, and Tandojam were tested for As^3+^ assay via the developed sensor based on Rano-Cu NPs. Samples were processed as per the described method in the experimental section. The linear plot (Figure 5b) was used for the measurement of As^3+^ in groundwater samples. The As^3+^ values were calculated and found to be in the range of 4.92–6.70 μM, with the recovery range from 97.96% to 102.5%, as presented in Table 2. This test highly supports our developed sensor for As^3+^ ions for each type of water sample in our study.

## 4. Conclusions

Highly stable ranolazine-capped Cu NPs were successfully synthesized under the reducing influence of hydrazine, without the assistance of an inert gas, via a one-pot chemical protocol. Characterization studies confirmed the formation of pure spherical particles with porous nature and crystalline properties. From a practical viewpoint, the presented synthetic method for Cu NPs is rapid, cheap, and simple to follow. The obtained Rano-Cu NPs were demonstrated to be extremely sensitive and highly selective as a colorimetric sensor for an exceptionally toxic ion, As^3+^, which is a global threat due to its presence in various water resources. The colorimetric sensor developed in this study, which relies on the preferential aggregation of Cu NPs by As^3+^ ions, is highly economical and may be useful for environmental protection agencies. To the best of our knowledge, this is the first report of using Cu NPs for colorimetric sensing of As^3+^. Its successful application in environmental groundwater samples further verifies and validates its practicality.

## Figures and Tables

**Table 1:** Comparison of reported methods with ranolazine-functionalized Cu NP sensor for detection of As^3+^.

Methods	Linear Range (M)	Limit of Detection (M)	Need for Addition of Surface Modification	Reference
Electrochemical method, graphene oxide	6.67 × 10^−5^ to 6.67 × 10^−4^	6.7 × 10^−6^	Yes	[38]
Bioassay, GFP-bb	1.0 × 10^−6^ to 2.5 × 10^−5^	4.0 × 10^−7^	Yes	[39]
Bioassay, GOx/Co_3_O_4_/GCE	6.67 × 10^−6^ to 1.4 × 10^−4^	5 × 10^−8^	Yes	[40]
Fluorescence	5.00 × 10^−6^ to 2.50 × 10^−4^	2.00 × 10^−8^	Yes	[41]
Colorimetric assay, Au NPs	2.67 × 10^−8^ to 2.67 × 10^−7^	3.34 × 10^−8^	Yes	[2]
Colorimetric assay, Au NPs	6.67 × 10^−8^ to 4.00 × 10^−5^	7.07 × 10^−8^	Yes	[42]
Colorimetric assay, Au NPs	5.34 × 10^−8^ to 1.33 × 10^−6^	2.4 × 10^−8^	No	[43]
Colorimetric assay, Rano-Cu NPs	3.0 × 10^−7^ to 8.3 × 10^−6^	1.6 × 10^−8^	No	Present work

GFP-bb: Green fluorescent protein-based bacteria.

**Table 2:** Determination of As^3+^ in real ground water samples.

Sample No.	As^3+^ Added (µM)	As^3+^ Recovered (μM)	Recovery (%)
1	4.8	4.92 ± 0.12	102.5
2	5.6	5.5 ± 0.13	97.69
3	6.6	6.70 ± 0.10	101.5

Condition = 3 mL Rano-Cu NP solution, number of replications = 3, and reaction time = 5 min; water sample 1, 2, and 3 from Jamshoro, Hala, and Tandojam, respectively.

## Supplementary Materials

The following are available online at http://www.mdpi.com/2079-4991/9/1/83/s1, Figure S1: Optimization studies for formation of Rano-Cu NPs, Figure S2: FTIR spectra of pure ranolazine and ranolazine-functionalized Cu NPs, Figure S3: XRD patterns of Rano-Cu NPs.

## References

[1] Cullen W.R., Reimer K.J. (1989). Arsenic speciation in the environment. Chem. Rev..

[2] Kalluri J.R., Arbneshi T., Khan S.A., Neely A., Candice P., Varisli B., Washington M., McAfee S., Robinson B., Banerjee S. (2009). Use of gold nanoparticles in a simple colorimetric and ultrasensitive dynamic light scattering assay: Selective detection of arsenic in groundwater. Angew. Chem..

[3] Xie X., Stueben D., Berner Z. (2005). The application of microelectrodes for the measurements of trace metals in water. Anal. Lett..

[4] Vaishanav S.K., Korram J., Pradhan P., Chandraker K., Nagwanshi R., Ghosh K.K., Satnami M.L. (2017). Green luminescent CdTe quantum dot based fluorescence nano-sensor for sensitive detection of arsenic (III). J. Fluor..

[5] Dai X., Compton R.G. (2006). Direct electrodeposition of gold nanoparticles onto indium tin oxide film coated glass: Application to the detection of arsenic (III). Anal. Sci..

[6] Chowdhury T., Zhang L., Zhang J., Aggarwal S. (2018). Removal of arsenic (III) from aqueous solution using metal organic framework-graphene oxide nanocomposite. Nanomaterials.

[7] Smith A.H., Hopenhayn-Rich C., Bates M.N., Goeden H.M., Hertz-Picciotto I., Duggan H.M., Wood R., Kosnett M.J., Smith M.T. (1992). Cancer risks from arsenic in drinking water. Environ. Health Perspect..

[8] Jena B.K., Raj C.R. (2008). Gold nanoelectrode ensembles for the simultaneous electrochemical detection of ultra trace arsenic, mercury, and copper. Anal. Chem..

[9] Sounderajan S., Udas A., Venkataramani B. (2007). Characterization of arsenic (V) and arsenic (III) in water samples using ammonium molybdate and estimation by graphite furnace atomic absorption spectroscopy. J. Hazard. Mater..

[10] Pantuzzo F.L., Silva J.C.J., Ciminelli V.S. (2009). A fast and accurate microwave-assisted digestion method for arsenic determination in complex mining residues by flame atomic absorption spectrometry. J. Hazard. Mater..

[11] Gil R., Ferrua N., Salonia J., Olsina R., Martinez L. (2007). On-line arsenic co-precipitation on ethyl vinyl acetate turning-packed mini-column followed by hydride generation-ICP OES determination. J. Hazard. Mater..

[12] Dufailly V., Noël L., Guérin T. (2008). Optimization and critical evaluation of a collision cell technology ICP-MS system for the determination of arsenic in foodstuffs of animal origin. Anal. Chim. Acta.

[13] Al-Assaf K.H., Tyson J.F., Uden P.C. (2009). Determination of four arsenic species in soil by sequential extraction and high performance liquid chromatography with post-column hydride generation and inductively coupled plasma optical emission spectrometry detection. J. Anal. At. Spectrosc..

[14] Nath P., Arun R.K., Chanda N. (2014). A paper based microfluidic device for the detection of arsenic using a gold nanosensor. RSC Adv..

[15] Ghorbani H.R. (2014). Chemical synthesis of copper nanoparticles. Orient. J. Chem..

[16] Rycenga M., Cobley C.M., Zeng J., Li W., Moran C.H., Zhang Q., Qin D., Xia Y. (2011). Controlling the synthesis and assembly of silver nanostructures for plasmonic applications. Chem. Rev..

[17] Jain P.K., Huang X., El-Sayed I.H., El-Sayed M.A. (2008). Noble metals on the nanoscale: Optical and photothermal properties and some applications in imaging, sensing, biology, and medicine. Acc. Chem. Res..

[18] Hussain I., Graham S., Wang Z., Tan B., Sherrington D.C., Rannard S.P., Cooper A.I., Brust M. (2005). Size-controlled synthesis of near-monodisperse gold nanoparticles in the 1–4 nm range using polymeric stabilizers. J. Am. Chem. Soc..

[19] Jian-Guang Y., Yuang-Lin Z., Okamoto T., Ichino R., Okido M. (2007). A new method for preparing hydrophobic nano-copper powders. J. Mat. Sci..

[20] Ahmed K.B.A., Sengan M., Kumar S., Veerappan A. (2016). Highly selective colorimetric cysteine sensor based on the formation of cysteine layer on copper nanoparticles. Sens. Actuators B Chem..

[21] El-Nour K.M.A., Eftaiha A.A., Al-Warthan A., Ammar R.A. (2010). Synthesis and applications of silver nanoparticles. Arab. J. Chem..

[22] Thakkar K.N., Mhatre S.S., Parikh R.Y. (2010). Biological synthesis of metallic nanoparticles. Nanomed. Nanotechnol. Biol. Med..

[23] Sun S., Kong C., Deng D., Song X., Ding B., Yang Z. (2011). Nanoparticle-aggregated octahedral copper hierarchical nanostructures. CrystEngComm.

[24] Ghosh S.K., Pal T. (2007). Interparticle coupling effect on the surface plasmon resonance of gold nanoparticles: From theory to applications. Chem. Rev..

[25] Noguez C. (2007). Surface plasmons on metal nanoparticles: The influence of shape and physical environment. J. Phys. Chem. C.

[26] Eustis S., El-Sayed M.A. (2006). Why gold nanoparticles are more precious than pretty gold: Noble metal surface plasmon resonance and its enhancement of the radiative and nonradiative properties of nanocrystals of different shapes. Chem. Soc. Rev..

[27] Wu S.-H., Chen D.-H. (2004). Synthesis of high-concentration Cu nanoparticles in aqueous CTAB solutions. J. Colloid Interface Sci..

[28] Al-Mamun M.A., Kusumoto Y., Muruganandham M. (2009). Simple new synthesis of copper nanoparticles in water/acetonitrile mixed solvent and their characterization. Mater. Lett..

[29] Park B.K., Jeong S., Kim D., Moon J., Lim S., Kim J.S. (2007). Synthesis and size control of monodisperse copper nanoparticles by polyol method. J. Colloid Interface Sci..

[30] De S., Mandal S. (2013). Surfactant-assisted shape control of copper nanostructures. Colloid Surf. A Physicochem. Eng. Asp..

[31] Hatamie A., Zargar B., Jalali A. (2014). Copper nanoparticles: A new colorimetric probe for quick, naked-eye detection of sulfide ions in water samples. Talanta.

[32] Ramajo L., Parra R., Reboredo M., Castro M. (2009). Preparation of amine coated silver nanoparticles using triethylenetetramine. J. Chem. Sci..

[33] Hussain M., Nafady A., Sherazi S.T.H., Shah M.R., Alsalme A., Kalhoro M.S., Mahesar S.A., Siddiqui S. (2016). Cefuroxime derived copper nanoparticles and their application as a colorimetric sensor for trace level detection of picric acid. RSC Adv..

[34] Cai Z.-X., Yang H., Zhang Y., Yan X.-P. (2006). Preparation, characterization and evaluation of water-soluble L-cysteine-capped-CdS nanoparticles as fluorescence probe for detection of Hg (II) in aqueous solution. Anal. Chim. Acta.

[35] Wang L.N., Li M.Y., Zeng Q.X. (2013). Synthesis of copper nanoparticles by chemical reduction method and its influential factors. Adv. Mater. Res..

[36] Gu X., Nguyen T., Oudina M., Martin D., Kidah B., Jasmin J., Rezig A., Sung L., Byrd E., Martin J.W. (2005). Microstructure and morphology of amine-cured epoxy coatings before and after outdoor exposures—An AFM study. JCT Res..

[37] Bothra S., Solanki J.N., Sahoo S.K. (2013). Functionalized silver nanoparticles as chemosensor for pH, Hg^2+^ and Fe^3+^ in aqueous medium. Sens. Actuators B Chem..

[38] Vasimalai N., Sheeba G., John S.A. (2012). Ultrasensitive fluorescence-quenched chemosensor for Hg (II) in aqueous solution based on mercaptothiadiazole capped silver nanoparticles. J. Hazard. Mater..

[39] Kumar S., Bhanjana G., Dilbaghi N., Kumar R., Umar A. (2016). Fabrication and characterization of highly sensitive and selective arsenic sensor based on ultra-thin graphene oxide nanosheets. Sens. Actuators B Chem..

[40] Liao V.H.C., Ou K.L. (2005). Development and testing of a green fluorescent protein-based bacterial biosensor for measuring bioavailable arsenic in contaminated groundwater samples. Environ. Toxicol. Chem..

[41] Wang X., Lv Y., Hou X. (2011). A potential visual fluorescence probe for ultratrace arsenic (III) detection by using glutathione-capped CdTe quantum dots. Talanta.

[42] Wu Y., Zhan S., Wang F., He L., Zhi W., Zhou P. (2012). Cationic polymers and aptamers mediated aggregation of gold nanoparticles for the colorimetric detection of arsenic (III) in aqueous solution. Chem. Commun..

[43] Gong L., Du B., Pan L., Liu Q., Yang K., Wang W., Zhao H., Wu L., He Y. (2017). Colorimetric aggregation assay for arsenic (III) using gold nanoparticles. Microchim. Acta.

