# Supplementary material for: Ranolazine-Functionalized Copper Nanoparticles as a Colorimetric Sensor for Trace Level Detection of As3+
Source: Nanomaterials (Basel). 2019 Jan 10;9(1):83. doi: 10.3390/nano9010083 (PMC6359034; doi:10.3390/nano9010083)
Supplement: Supplementary file 1 [file nanomaterials-09-00083-s001.pdf]

Supplementary Material

Ranolazine-Functionalized Copper Nanoparticles as a Colorimetric Sensor for Trace Level Detection of As^{3+}

Gul Naz Laghari [1], Ayman Nafady [2,3,*], Sameerah I. Al-Saeedi [4], Sirajuddin [1,*], Syed Tufail H. Sherazi [1], Jan Nisar [5], Muhammad Raza Shah [6], Mohammad I. Abro [7], Munazza Arain [8] and Suresh K. Bhargava [9]

[1] National Centre of Excellence in Analytical Chemistry, University of Sindh, Jamshoro 76080, Pakistan; gulnaz.laghari@yahoo.com (G.N.L.); tufail.sherazi@gmail.com (S.T.H.S.)
[2] Department of Chemistry, College of Science, King Saud University, Riyadh 11451, Saudi Arabia
[3] Department of Chemistry, Faculty of Science, Sohag University, Sohag 82524, Egypt
[4] Department of Chemistry, College of Science, Princess Nourah bint Abdulrahman University, Riyadh 11451, Saudi Arabia; sialsaeedi@pnu.edu.sa
[5] National Centre of Excellence in Physical Chemistry, University of Peshawar, Peshawar 25120, Pakistan; pashkalawati@gmail.com
[6] International Centre of Chemical and Biological Science, HEJ Research Institute of Chemistry, University of Karachi, Karachi 75270, Pakistan; raza.shah@iccs.edu
[7] Department of Metallurgy and Materials Engineering, Mehran University of Engineering & Technology, Jamshoro 76080, Pakistan; m_ishaqueabro@yahoo.com
[8] Dr. MA Kazi Institute of Chemistry, University of Sindh, Jamshoro 76080, Pakistan; munazzaarain2493@gmail.com
[9] Centre for Advanced Materials and Industrial Chemistry (CAMIC), School of Applied Sciences, RMIT University, GPO BOX 2476, Melbourne 3001, Australia; suresh.bhargava@rmit.edu.au
* Correspondence: anafady2004@yahoo.com or anafady@ksu.edu.sa (A.N.); drsiraj03@yahoo.com (S.); Tel.: +966-569407110 (A.N.); +92-9213429 (S.); Fax: +92-9213431 (S.)

Table of Content

Figure S1(a): Optimization study of precursor Cu salt ranging from 100 μL to 450 μL.

Figure S1(b): Optimization study of reducing agent in the range of 100 to 600 μL

Figure S1(c): Optimization study of ranolazine ranging from 100 to 600 μL

Figure S1(d): Optimization study showing formation of Cu NPs at pH value ranging from 5 to 11

Figure S2: FTIR spectra of pure ranolazine (black curve) and ranolazine-functionalized Cu NPs (red curve).

Figure S3: PXRD patterns of ranolazine copper nanoparticles

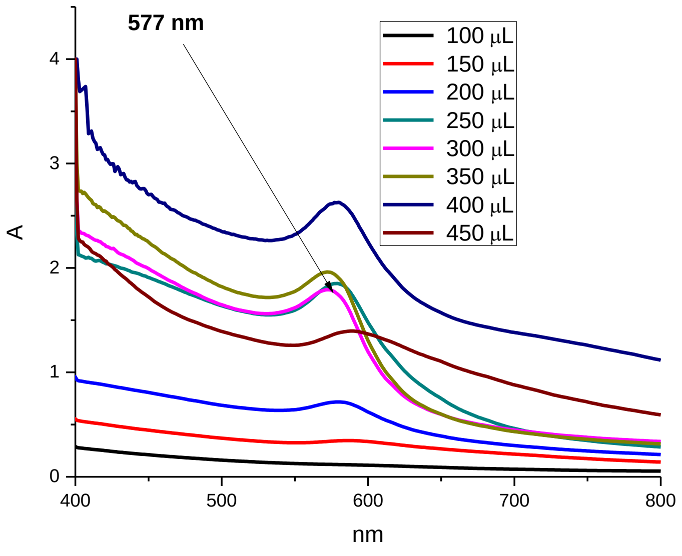

Figure S1(a): Optimization study of precursor Cu salt ranging from 100 μL to 450 μL.

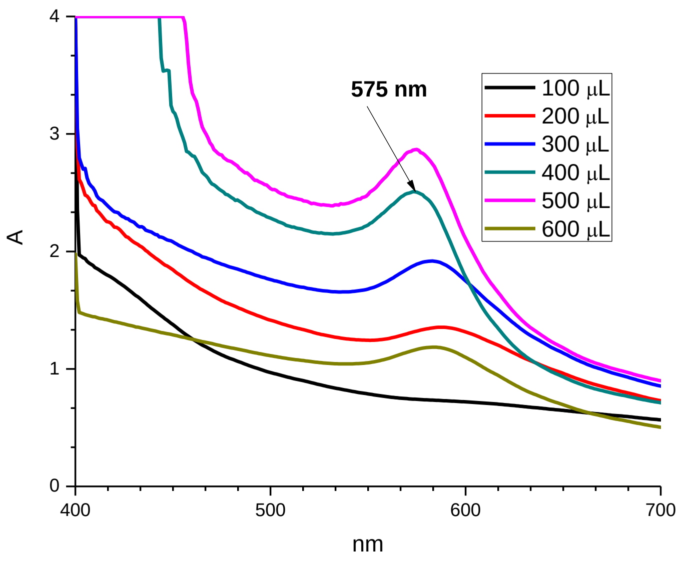

Figure S1(b): Optimization study of reducing agent in the range of 100 to 600 μL.

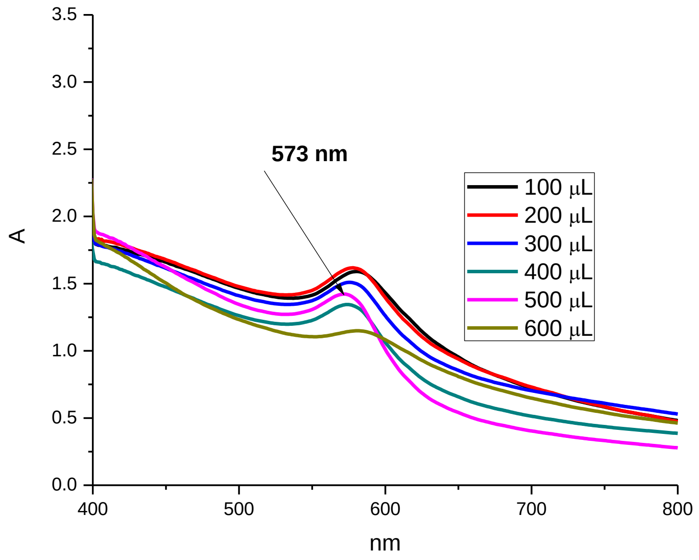

Figure S1(c): Optimization study of ranolazine ranging from 100 to 600 μL**.**

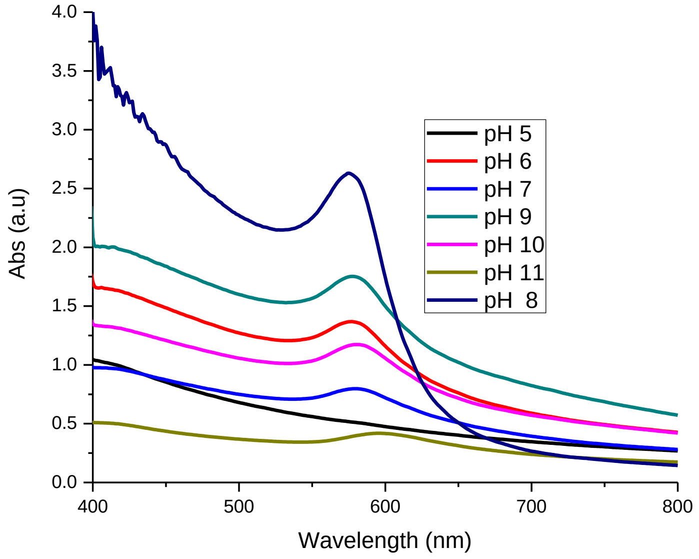

Figure S1(d): Optimization study showing formation of Cu NPs at pH value ranging from 5 to 11.

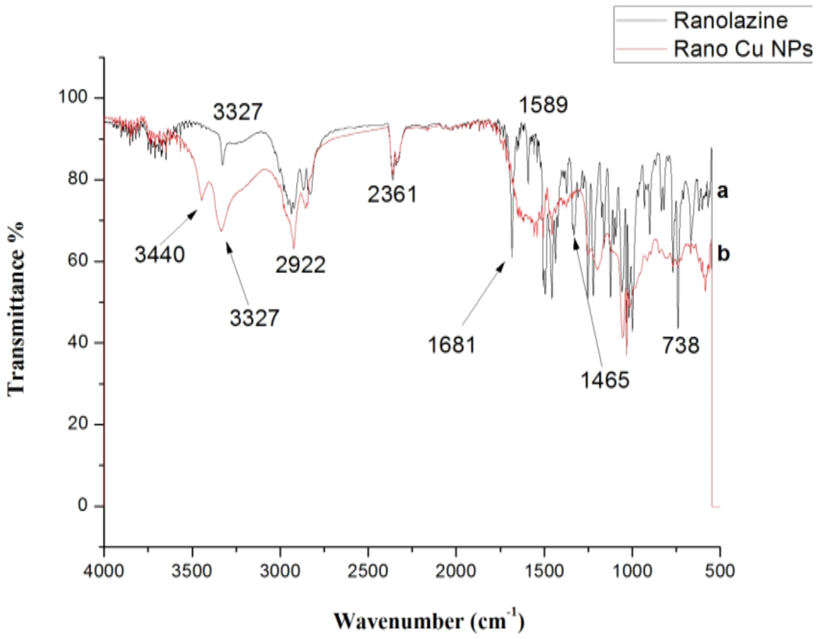

Figure S2: FTIR spectra of pure ranolazine (black curve) and ranolazine-functionalized Cu NPs (red curve).

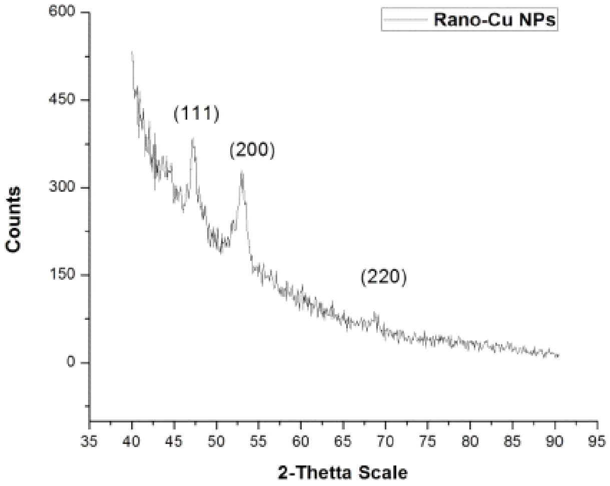

Figure S3. PXRD patterns of ranolazine copper nanoparticles.
